# Metagenome Assembly and Metagenome-Assembled Genome Sequences from the Rhizosphere of Maize Plants in Mafikeng, South Africa

**DOI:** 10.1128/MRA.00954-20

**Published:** 2021-02-25

**Authors:** Olubukola O. Babalola, Rebaona R. Molefe, Adenike E. Amoo

**Affiliations:** a Food Security and Safety Niche, Faculty of Natural and Agricultural Sciences, North-West University, Mmabatho, South Africa; Georgia Institute of Technology

## Abstract

The rhizosphere microbiome plays an essential role in enhancing the growth of plants, raising the need for comprehension of their metabolic abilities. Here, we investigated rhizospheric and bulk soils of maize plants in Mafikeng, South Africa. Metagenome-assembled genomes containing plant growth-promoting genes were reconstructed.

## ANNOUNCEMENT

Zea mays (maize) has become one of the foremost staple crops in modern agriculture, with roughly 1.4 billion tonnes produced annually in 2017. Organic compounds, including amino acids, sugars, and mucilage, are exuded by the roots of maize plants, thereby attracting microbes from the bulk soil to the rhizosphere. Rhizosphere microbes can have significant influences on plant growth; plant growth-promoting rhizobacteria (PGPR) have been proposed as an ecological additive to enhance plant growth (1–3). It is therefore imperative to identify functional genes that typify the rhizospheric microbes of maize for a proper understanding of the metabolic capabilities of the rhizosphere.

Replicate soil samples were collected from the rhizosphere (F4R1 and F4R2) of maize plants and the bulk soil (F4B1 and F4B2) from a farm situated in Mafikeng, South Africa (25°85′S, 25°63′33″E). The rhizosphere soil samples were collected on 17 June 2019 at 8-cm diameter and 15-cm depth of the maize plants. The soil samples were transported to the laboratory on ice and stored until further use. Genomic DNA extraction was carried out using the DNeasy PowerSoil DNA isolation kit (MoBio Laboratories, Carlsbad, CA) in accordance with the manufacturer’s directions. Using 50 ng of DNA from each sample, libraries were prepared using the Nextera DNA Flex library preparation kit (Illumina) as instructed by the manufacturer. The determination of library average insert size was done using the Agilent 2100 bioanalyzer. The libraries were pooled, diluted (to 0.6 nM), and sequenced as paired ends for 300 cycles using the NovaSeq system (Illumina).

The sequencing results are shown in Table 1. The metagenomic sequences were analyzed using the DOE Systems Biology Knowledgebase (KBase) (4). Except where otherwise stated, default parameters were used for all the software employed. For the processing of the paired-end reads, the qualities of the read libraries were examined with FastQC v0.11.5 (5), and Trimmomatic v0.36 (6) was used for the elimination of barcode sequences and sections of truncated quality from the reads. Combined assemblies were generated (F4R1 and F4R2 formed F4R, while F4B1 and F4B2 formed F4B). The metagenomes were assembled into contigs using MEGAHIT v1.2.9 (7), and coverage information was determined using Bowtie 2 v2.3.2 (8). Metagenome-assembled genomes (MAGs) were reconstructed with MaxBin 2 v2.2.4 (9). The quality of the MAGs was determined using CheckM v1.0.18 (10). Using GTDB-Tk classification, the MAGs were classified taxonomically. For F4R, a MAG classified as a member of the *Actinobacteriota* was generated. One MAG assigned to an unclassified taxon was reconstructed for F4B. The RAST algorithm (11) was applied for annotating the genomes, and standard features were called using Glimmer3 and Prodigal. Using Kmers v2, Kmers v1, and protein similarity, the genome features were functionally annotated. The functional analysis showed that the resulting MAGs have genes involved in plant growth promotion. The MAG was classified as *Actinobacteria*, a class that has been reported to possess plant growth-promoting traits (12).

**TABLE 1:** Genome statistics of the maize rhizosphere and bulk soil metagenomes

Genome characteristic	Data for sample code:			
	F4R1	F4R2	F4B1	F4B2
SRA accession no.	SRR12285195	SRR12285194	SRR12285196	SRR12285197
Raw read counts				
No. of reads (paired-end)	26,868,696	20,420,846	25,092,166	20,222,698
Total no. of bases	3,467,131,872	2,641,232,068	3,121,851,578	2,660,693,984
GC content (%)	64.53	64.23	65.04	64.93
Counts post-QC (no. of reads retained)	12,906,831	9,730,631	11,942,839	19,336,030
	**F4R**		**F4B**	
Counts after combined assembly formation				
No. of contigs	1,570		241	
Avg length (bp)	2,471.75		2,388.99	
Coverage estimate	7.54 ± 9.85		7.47 ± 12.06	
*N*_50_ (bp)	2,390		2,280	

### Data availability.

The raw sequencing data are available at the NCBI Sequence Read Archive (SRA) under BioProject PRJNA647806 with accession numbers SRR12285195 (F4R1), SRR12285194 (F4R2), SRR12285196 (F4B1), and SRR12285197 (F4B2). The metagenome assemblies and the MAGs are available at the European Nucleotide Archive (ENA) under the project number PRJEB39783 with accession numbers SAMEA7159858 (F4R assembly), SAMEA7819642 (F4B assembly), SAMEA7819643 (F4R MAG), and SAMEA7819640 (F4B MAG).

## References

[1] Beirinckx S, Viaene T, Haegeman A, Debode J, Amery F, Vandenabeele S, Nelissen H, Inzé D, Tito R, Raes J, De Tender C, Goormachtig S. 2020. Tapping into the maize root microbiome to identify bacteria that promote growth under chilling conditions. Microbiome 8:1–13. doi:10.1186/s40168-020-00833-w. 32305066 PMC7166315

[2] Babalola OO. 2010. Beneficial bacteria of agricultural importance. Biotechnol Lett 32:1559–1570. doi:10.1007/s10529-010-0347-0. 20635120

[3] Brisson VL, Schmidt JE, Northen TR, Vogel JP, Gaudin AC. 2019. Impacts of maize domestication and breeding on rhizosphere microbial community recruitment from a nutrient depleted agricultural soil. Sci Rep 9:14. doi:10.1038/s41598-019-52148-y. 31666614 PMC6821752

[4] Arkin AP, Cottingham RW, Henry CS, Harris NL, Stevens RL, Maslov S, Dehal P, Ware D, Perez F, Canon S, Sneddon MW, Henderson ML, Riehl WJ, Murphy-Olson D, Chan SY, Kamimura RT, Kumari S, Drake MM, Brettin TS, Glass EM, Chivian D, Gunter D, Weston DJ, Allen BH, Baumohl J, Best AA, Bowen B, Brenner SE, Bun CC, Chandonia J-M, Chia J-M, Colasanti R, Conrad N, Davis JJ, Davison BH, DeJongh M, Devoid S, Dietrich E, Dubchak I, Edirisinghe JN, Fang G, Faria JP, Frybarger PM, Gerlach W, Gerstein M, Greiner A, Gurtowski J, Haun HL, He F, Jain R, Joachimiak MP, Keegan KP, Kondo S, Kumar V, Land ML, Meyer F, Mills M, Novichkov PS, Oh T, Olsen GJ, Olson R, Parrello B, Pasternak S, Pearson E, Poon SS, Price GA, Ramakrishnan S, Ranjan P, Ronald PC, Schatz MC, Seaver SMD, Shukla M, Sutormin RA, Syed MH, Thomason J, Tintle NL, Wang D, Xia F, Yoo H, Yoo S, Yu D. 2018. KBase: the United States Department of Energy Systems Biology Knowledgebase. Nat Biotechnol 36:566–569. doi:10.1038/nbt.4163. 29979655 PMC6870991

[5] Andrews S. 2010. FastQC: a quality control tool for high throughput sequence data. https://www.bioinformatics.babraham.ac.uk/projects/fastqc/.

[6] Bolger AM, Lohse M, Usadel B. 2014. Trimmomatic: a flexible trimmer for Illumina sequence data. Bioinformatics 30:2114–2120. doi:10.1093/bioinformatics/btu170. 24695404 PMC4103590

[7] Li D, Liu C-M, Luo R, Sadakane K, Lam T-W. 2015. MEGAHIT: an ultra-fast single-node solution for large and complex metagenomics assembly via succinct de Bruijn graph. Bioinformatics 31:1674–1676. doi:10.1093/bioinformatics/btv033. 25609793

[8] Langmead B, Salzberg S. 2012. Fast gapped-read alignment with Bowtie 2. Nat Methods 9:357–359. doi:10.1038/nmeth.1923. 22388286 PMC3322381

[9] Wu Y-W, Tang Y-H, Tringe SG, Simmons BA, Singer SW. 2014. MaxBin: an automated binning method to recover individual genomes from metagenomes using an expectation-maximization algorithm. Microbiome 2:26. doi:10.1186/2049-2618-2-26. 25136443 PMC4129434

[10] Parks DH, Imelfort M, Skennerton CT, Hugenholtz P, Tyson GW. 2015. CheckM: assessing the quality of microbial genomes recovered from isolates, single cells, and metagenomes. Genome Res 25:1043–1055. doi:10.1101/gr.186072.114. 25977477 PMC4484387

[11] Brettin T, Davis JJ, Disz T, Edwards RA, Gerdes S, Olsen GJ, Olson R, Overbeek R, Parrello B, Pusch GD, Shukla M, Thomason JA, Stevens R, Vonstein V, Wattam AR, Xia F. 2015. RASTtk: a modular and extensible implementation of the RAST algorithm for building custom annotation pipelines and annotating batches of genomes. Sci Rep 5:8365. doi:10.1038/srep08365. 25666585 PMC4322359

[12] Sathya A, Vijayabharathi R, Gopalakrishnan S. 2017. Plant growth-promoting actinobacteria: a new strategy for enhancing sustainable production and protection of grain legumes. 3 Biotech 7:1–10. doi:10.1007/s13205-017-0736-3. PMC5449283 28560641

